# Exploring the Role of Spatial Frequency Information during Neural Emotion Processing in Human Infants

**DOI:** 10.3389/fnhum.2017.00486

**Published:** 2017-10-09

**Authors:** Sarah Jessen, Tobias Grossmann

**Affiliations:** ^1^Research Group “Early Social Development”, Max Planck Institute for Human Cognitive and Brain Sciences, Leipzig, Germany; ^2^Department of Neurology, University of Lübeck Lübeck, Germany; ^3^Department of Psychology, University of Virginia, Charlottesville, VA, United States

**Keywords:** infancy, face processing, emotion perception, EEG, spatial frequencies

## Abstract

Enhanced attention to fear expressions in adults is primarily driven by information from low as opposed to high spatial frequencies contained in faces. However, little is known about the role of spatial frequency information in emotion processing during infancy. In the present study, we examined the role of low compared to high spatial frequencies in the processing of happy and fearful facial expressions by using filtered face stimuli and measuring event-related brain potentials (ERPs) in 7-month-old infants (*N* = 26). Our results revealed that infants’ brains discriminated between emotional facial expressions containing high but not between expressions containing low spatial frequencies. Specifically, happy faces containing high spatial frequencies elicited a smaller Nc amplitude than fearful faces containing high spatial frequencies and happy and fearful faces containing low spatial frequencies. Our results demonstrate that already in infancy spatial frequency content influences the processing of facial emotions. Furthermore, we observed that fearful facial expressions elicited a comparable Nc response for high and low spatial frequencies, suggesting a robust detection of fearful faces irrespective of spatial frequency content, whereas the detection of happy facial expressions was contingent upon frequency content. In summary, these data provide new insights into the neural processing of facial emotions in early development by highlighting the differential role played by spatial frequencies in the detection of fear and happiness.

## Introduction

Fast and efficient processing of emotional information is crucial for human behavior as it enables adaptive responding during social interactions (Frith, 2009). Over the past two decades, much research has focused on investigating the neural basis of emotion processing (Adolphs, 2002; Güntekin and Başar, 2014; de Gelder et al., 2015; Kragel and LaBar, 2016). One important insight from this area of research is that magno- and parvocellular pathways in the visual system contribute in different ways to emotion processing. In particular, there is work to show that fast and efficient emotion processing is predominantly instantiated by the magnocellular pathway, whereas more detailed processing of facial information primarily involves the parvocellular pathway (Vuilleumier et al., 2003).

The general properties of these two pathways have been intensively studied in vision research. Independent of emotional content, the magnocellular pathway is primarily responsible for the fast, yet coarse, processing of visual input, while the parvocellular pathway is mainly involved in the slower processing of fine visual details (Livingstone and Hubel, 1988). The two pathways can be studied by filtering the visual input with respect to its spatial frequency information (Hammarrenger et al., 2003). Specifically, while the parvocellular pathway is most sensitive to high spatial frequency (HSF) information, the magnocellular pathway is most sensitive to low spatial frequencies (LSF; Vuilleumier et al., 2003). LSF filtered images predominantly contain global information, while HSF filtered images provide more detailed information necessary for fine-grained processing of images (Goffaux and Rossion, 2006).

In recent years, the differential processing of HSF and LSF has been used to study different aspects of visual emotion processing in human adults. In particular, while LSF information (<6 cycles/°) appears to play a crucial role in the detection and classification of fearful information, HSF information (>24 cycles/°) is more important for non-emotional face processing such as facial identity matching (Vuilleumier et al., 2003). Accordingly, in adults, activity in the fusiform cortex is mainly driven by the HSF content of images, while activity in the amygdala is primarily driven by the LSF content of images (Vuilleumier et al., 2003; Méndez-Bértolo et al., 2016). It has been suggested that emotionally negative LSF input primarily activates the magnocellular pathway which in turn elicits a fast and efficient processing of highly salient and arousing information in the amygdala (Vuilleumier et al., 2003). In contrast, the observed activation of the fusiform cortex by viewing HSF images points to a slow and more detailed processing of facial features required for identity recognition (Vuilleumier et al., 2003). Moreover, the predominant processing of emotionally salient information via a subcortical pathway receiving mainly magnocellular input has also been argued to underpin non-conscious visual emotion processing (for a review, see Tamietto and de Gelder, 2010). For example, cortically blind patients show sensitive responding to fearful information, which is thought to rely on a subcortical pathway bypassing cortical visual processing (de Gelder et al., 1999).

Further evidence for a specific role of the magnocellular pathway in the fast processing of emotionally salient information comes from event-related brain potential (ERP) studies. Viewing LSF filtered images of fearful faces but not HSF filtered images result in an enhancement of the visual P1 in adults (Pourtois et al., 2005; Vlamings et al., 2009). The P1 originates from the extrastriate visual cortex and is an ERP peaking between 100 ms and 130 ms post-stimulus in response to particularly salient visual information (Clark and Hillyard, 1996), indicating an increased allocation of attention to LSF filtered images of fearful faces (Pourtois et al., 2005). Although the P1 occurs before the N170 component, which is commonly linked to the structural processing of faces (Rossion, 2014), a number of studies report a modulation of P1 amplitude by emotional, in particular fearful, facial expressions (Batty and Taylor, 2003; Pourtois et al., 2004; Smith et al., 2013). The fact that emotional content can modulate brain responses before the structural processing of facial information takes place provides further support for the existence of a fast but coarse pathway that bypasses classical face processing to elicit a rapid response to negative, in particular fearful, facial expressions.

While the evidence from work with adults supports the notion of fast responding to fearful facial expressions mediated via LSF and the magnocellular pathway, little is known about the role of the magnocellular and parvocellular pathways in emotion processing in development. Recent findings suggest that children rely on HSF rather than LSF information when detecting fearful facial expressions (Vlamings et al., 2010). Specifically, Vlamings et al. (2010) recorded EEG responses from children between 3 and 8 years of age in response to HSF and LSF fearful and neutral facial expressions. In contrast to previous findings with adults, children showed an enhanced P1 for fearful compared to neutral faces only when HSF images were presented. This is taken to suggest that children rely on different frequency information and might need more detailed feature-focused information than adults when processing fearful facial expressions. This developmental view has been confirmed by a recent ERP study with 9-to-10-month-old infants showing that infants at this age also predominantly use HSF to discriminate happy, fearful and neutral facial expressions (Munsters et al., 2017). Specifically, this study revealed differential processing of emotional facial expressions in response to HSF images but not for LSF images. Munsters et al. (2017) observed emotion-related differences for face-sensitive ERP components (N290/P400 complex) seen as precursors of the adult N170, reflecting the structural encoding of faces (Eimer, 2000; Rossion, 2014). Together, these two developmental ERP studies point to the notion that in infants and children HSF information is needed for facial emotion processing to occur.

However, emotion discrimination from faces, in particular involving fearful faces, typically affects additional ERP components and can be reliably observed already in infants at a younger age (Peltola et al., 2009, 2013) than in Munsters et al. (2017) study, who investigated emotion processing in 9- to 10-month-old infants. Moreover, spatial frequency filtering might need to be adjusted to take into account the visual acuity at the age under investigation (Dobkins and Harms, 2014), which had not been done in the prior study with infants that used spatial frequency cut-offs typically used with adults (Munsters et al., 2016). It thus remains unclear whether, similar to what is known from adults, infants rely on LSF when frequency cut-offs are adjusted to their visual acuity. We therefore decided to extend this line of research by studying infants at a younger age and by using age-appropriate spatial frequency filters for stimulus generation. In the following, we will provide a detailed rationale for our experimental approach.

By 7 months of age, infants develop an attentional bias towards fearful expressions, which manifests itself in prolonged looking duration to fearful faces when compared to happy faces and in enhanced ERP responses to fearful facial expressions (Vaish et al., 2008; Peltola et al., 2009). The Nc ERP component, a central negativity linked to attention allocation and localized to prefrontal and anterior cingulate cortex is of particular interest in this context (Webb et al., 2005). The Nc typically shows an enhanced amplitude in response to fearful compared to happy facial expressions (e.g., Peltola et al., 2009; Grossmann et al., 2011), but differential Nc responses can also be observed between different negative expressions, such as anger and fear (Kobiella et al., 2008). A modulation of the Nc amplitude cannot only be observed following conscious processing of emotional information but is also seen in the absence of conscious perception of facial cues (Jessen and Grossmann, 2014, 2015). Furthermore, the N290/P400 ERP complex, which has been discussed as a precursor of the face-specific adult N170 (de Haan et al., 2003), has also been shown to vary as a function of emotion in infants (Leppänen et al., 2007; Kobiella et al., 2008), which is similar to what has been observed in adults (Batty and Taylor, 2003; Blau et al., 2007; Pegna et al., 2008).

Recently, using ERPs and eyetracking it has been shown that infants detect fearful faces independent of conscious perception (Jessen and Grossmann, 2014, 2015; Jessen et al., 2016), a function that has been linked to the subcortical (magnocellular) processing route in adults (Whalen et al., 2004). These recent findings with infants thus suggest that infants’ emotion detection might rely on a subcortical route for face processing based on information received through the magnocellular system. If infants process fearful information predominantly via the magnocellular pathway, one would expect the same distinction in processing LSF images of fearful facial expressions but not HSF images as observed in adults (Vuilleumier et al., 2003; Pourtois et al., 2005; Méndez-Bértolo et al., 2016). Importantly, differential processing of subliminally presented emotional expressions has only been observed for the Nc but not for the P400 (Jessen and Grossmann, 2015). If unconscious (subliminal) emotion processing relies on the same pathway involving subcortical brain regions as the fast emotional responses elicited by images containing only LSF information (Tamietto and de Gelder, 2010), then it might be expected that differential processing of LSF images of emotional faces will predominantly effect the Nc response but not the P400.

In the current study, we presented 7-month-old infants with images of faces expressing fear or happiness, which were manipulated to contain predominately high or low spatial frequencies. One important issue to consider when studying the role of the magnocellular compared to the parvocellular system in emotion processing in infants is the protracted development of visual acuity in humans. Visual spatial acuity matures slowly, and an adult-like acuity can only be observed from around 6–7 years (Ellemberg et al., 1999). The same holds true for the processing of high compared to low spatial frequencies, which continues to develop throughout childhood (van den Boomen et al., 2015). Furthermore, it has been shown that at the structural (anatomical) level the magnocellular pathway matures faster than the parvocellular pathway (Hammarrenger et al., 2003). Thus, when investigating the processing of spatial frequencies and its influence on higher-level visual processing in a developmental population, it is important to differentiate between relative and absolute high and low spatial frequencies. While studies in adults typically assume a range of 6–24 cycles/image as the preferred range for face processing, and accordingly define HSF as >24 cycles/image and LSF as <6 cycles/image (Vuilleumier et al., 2003; Pourtois et al., 2005), this does not necessarily correspond to the visual acuity in infants and young children. While Munsters et al. (2017), who investigated the role of spatial frequencies in 9–10 month-olds, used 2 cycles/° as an upper boundary for their LSF images and 6 cycles/° as a lower boundary for their HSF images (Munsters et al., 2017), which corresponds to the frequency ranges previously used in adults (Munsters et al., 2016), other studies on the role of spatial frequency content in face processing in a developmental population have often adapted the frequency ranges to the assumed visual acuity at a given age. When adjusting the spatial frequencies contained in the stimulus material to the visual acuity of infants, it has been found that newborns process facial information primarily via spatial frequencies below 0.5 cycles/° (equivalent to 12 cycles/image; de Heering et al., 2008). However, more recent work with 8-month-old infants found a face-inversion effect only for HSF (above 0.6 cycles/° Dobkins and Harms, 2014) but not for LSF. Based on previous work using thresholds adapted to infant visual acuity, we therefore used 0.5 cycles/° as a cut-off point, which represents the spatial frequency most closely approximating the peak spatial frequency of the contrast sensitivity curve at 8 months of age (Peterzell, 1993; Dobkins and Harms, 2014). Thus, in the current study, LSF images contained frequencies below 0.4 cycles/° while HSF images contained frequencies above 0.6 cycles/°.

Based on previous studies that used unfiltered facial stimuli (Peltola et al., 2009; Jessen and Grossmann, 2016), we decided to study infants at the age of 7 months, because this is the age by which infants first show heightened allocation of attention to fearful faces in their looking time and ERPs. Critically, if infants use similar brain processes for fear detection to adults, involving the magnocellular system, then we would expect to see selective effects on processing fear from LSF faces but not necessarily from HSF faces. If, in contrast, infants rely predominantly on information from the parvocellular system, we expect a differential effect only for images containing high spatial frequencies. Addressing this question by examining the role of spatial frequency information in infants’ emotion processing fills an important gap in our understanding of the neurodevelopment of facial emotion processing systems.

## Materials and Methods

### Participants

Twenty six 7-month-old infants (mean age: 219 days, range: 205–230 days, 15 female) were included in the final sample. This sample size was determined* a priori* based on comparable ERP studies on emotion perception in infancy (Leppänen et al., 2007; Kobiella et al., 2008; Peltola et al., 2009). An additional three infants were tested but not included in the analysis because of failure to contribute at least 10 artifact-free trials per conditions (*N* = 2) or the mean amplitude across all conditions in the ROI and time-window used to analyze the Nc response was more than 2 standard deviations (SD) above or below the mean (*N* = 1). Infants contributed on average 35 ± 13 (mean ± SD) trials per condition (happy-LSF: 34 ± 14, happy-HSF: 35 ± 13, fear-LSF: 35 ± 13, fear-HSF: 36 ± 12).

All infants were born full-term (38–42 weeks gestational age) and had a birth-weight of at least 2500 g. This study was carried out in accordance with the recommendations of the ethics committee at the University of Leipzig with written informed consent from the parents of all subjects. All parents of all subjects gave written informed consent in accordance with the Declaration of Helsinki. The protocol was approved by the ethics committee at the University of Leipzig.

### Stimuli

**Figure 1:**
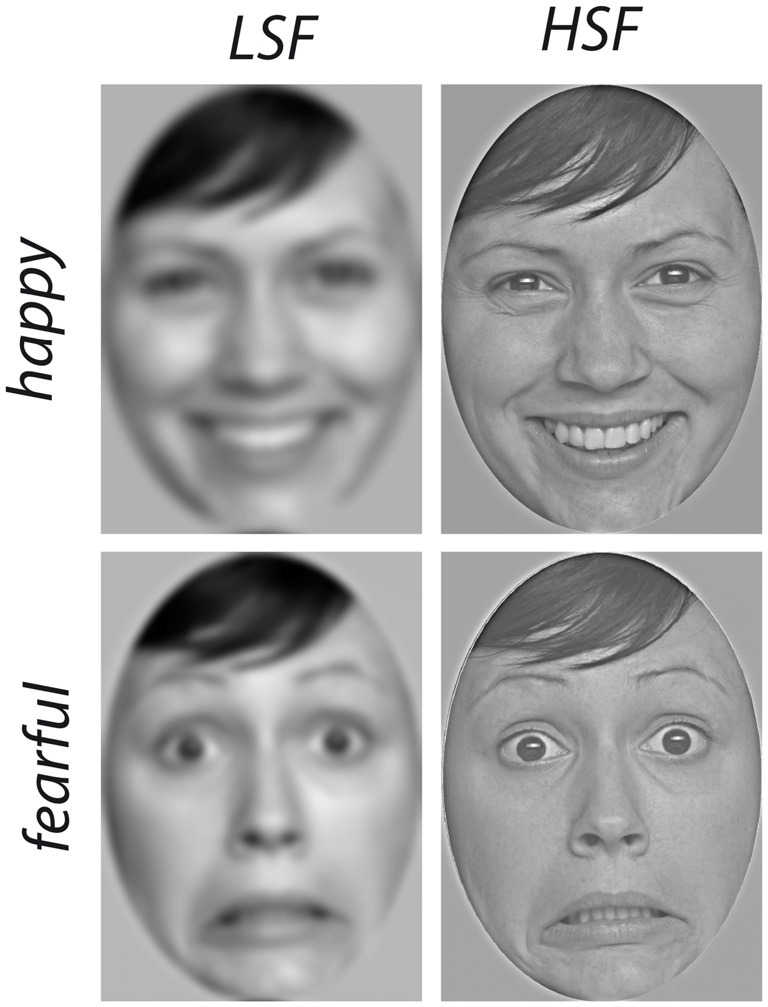
Examples of stimulus material. Images of happy (top row) and fearful (bottom row) faces were filtered to contain only spatial frequencies below 0.4 cycles/° (low spatial frequencies (LSF), left column) or above 0.6 cycles/° (high spatial frequencies (HSF), right column).

The stimulus material consisted of photographs of six different actresses from the FACES database (age 18–30, ID-number 54, 63, 85, 90, 115 and 173, see Ebner et al., 2010) expressing fear and happiness, see Figure 1 for an example. All images were edited according to an established procedure by transforming faces to gray-scale images and applying a spatial filter using a Matlab script adapted from Paul van Diepen^1^ (see Dobkins and Harms, 2014). For the stimulus images containing only HSF, a cut-off of 0.6 cycles/° (or 4.8 cycles/ face width) was chosen, while for the stimulus images containing LSF, a cut-off of 0.4 cycles/° (or 3.2 cycles/ face width) was used, based on the values used for a comparable age group by Dobkins and Harms (2014). The images did not differ in luminance (*p* > 0.4, as calculated based on the RGB values using Matlab) and had a standardized height of 18.5 cm and a width of 13 cm, leading to a horizontal visual angle of about 8° and a vertical visual angle of about 12° (at 90 cm viewing distance).

### Design

The experiment consisted of four conditions resulting in a 2 × 2 design with the factors Emotion (happy, fearful) and Frequency (HSF, LSF). Per condition, 84 trials were presented (14 per actress), leading to a total of 336 trials. The trials were presented in pseudo-randomized order, ensuring that the same condition was not presented more than twice in a row. Furthermore, trials were arranged into miniblocks consisting of 24 trials each (6 trials per condition, 1 trial per actress). The miniblocks were presented consecutively without interruption. Each participant received an individual randomization list. Every trial started with the presentation of a black fixation star on a gray background for 300 ms followed by the stimulus face presented for 750 ms. After each trial, a gray screen was shown for a randomly varying duration between 800 ms and 1200 ms.

### Procedure

After arrival in the lab, infants and parents were familiarized with the new environment, and parents were informed about the experiment and then signed a consent form. The EEG recording was prepared while the infant was sitting on his or her parent’s lap. An elastic cap (EasyCap) in which 27 Ag-Ag-Cl-electrodes were mounted according to the 10-20-system was used for recording. Additionally, an electrode was attached below the infant’s right eye for computing the electrooculogram (EOG). The EEG was recorded with a sampling rate of 500 Hz using a PORTI-32/MREFA amplifier (Twente Medical Systems). The Cz electrode was used as an online reference. The experiment took place in a soundproof, electrically shielded chamber, in which the infant was seated on his or her parent’s lap. Stimuli were presented on a CRT monitor with a screen resolution of 1024 × 786 and a refresh rate of 60 Hz at a distance of approximately 90 cm from the infant. The parent was instructed not to interact with the infant during the experiment. Infants’ looking behavior during the experiment was monitored using a small camera mounted on top of the monitor. When the infant became inattentive, video clips with colorful moving abstract shapes accompanied by ring tones were played in order to redirect the infant’s attention to the screen. The experiment continued until the maximum number of trials was presented or the infant became too fussy to continue the experiment.

### EEG Analysis

Data were re-referenced to the mean across all electrodes (average reference), and bandpass-filtered between 0.2 Hz and 20 Hz. Trials were segmented into 1 s-epochs lasting from 200 ms before stimulus onset to 800 m after stimulus onset. In five participants one electrode was noisy and therefore interpolated using spherical spline interpolation (Perrin et al., 1989). In order to detect trials contaminated by artifacts, the standard deviation was computed in a sliding window of 200 ms. If the standard deviation exceeded 80 μV at any electrode or in the EOG, the entire trials was discarded. Additionally, the trials were inspected visually to ensure no artifacts remained. Furthermore, the video recording of the infants during the experiments was analyzed and all trials in which the infant did not attend to the screen were excluded from further analysis. To analyze the Nc amplitude, data were averaged for each condition at frontal electrodes (F3, Fz, F4) in a time-window from 500 ms to 600 ms after stimulus onset. This time window was determined based on visual inspection of the resulting wave form in order to appropriately capture the peak of the Nc. To analyze the N290 and P400 amplitude, we averaged the data at O1, O2, P7 and P8 from 150 ms to 300 ms (N290) and 350 ms to 600 ms (P400) after stimulus onset. The mean amplitude in these time-windows was entered into a repeated-measures analysis of variance (ANOVA) with the factors Emotion (fearful, happy) and Frequency (HSF, LSF). Student’s *t-*tests were computed to further analyze interaction effects. Effect sizes are reported as partial eta-squared ($\eta_{\text{p}}^{2}$) for ANOVAs and *r* for *t-*tests.

## Results

### Nc

**Figure 2:**
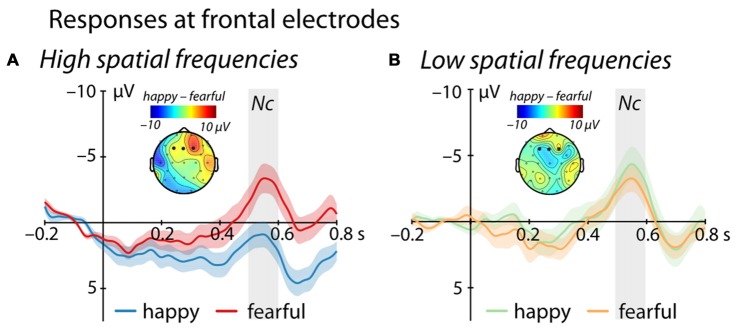
Event-related brain potential (ERP) response at frontal electrodes (F3, Fz, F4). **(A)** shows mean responses to images containing HSF while **(B)** displays responses to images containing LSF (blue/green = happy expression, red/orange = fearful expression; displayed are mean responses ± within-subject standard errors). Topographic representations show the difference in activation following happy and fearful faces between 500 ms and 600 ms, corresponding to the time-window used in the statistical analysis and marked in gray.

Between 500 ms and 600 ms after stimulus onset, we observed an interaction between Emotion and Frequency at frontal electrodes (*F*_(1,25)_ = 4.69, *p* = 0.04, $\eta_{\text{p}}^{2}$ = 0.16, see Figure 2). Specifically, for images containing only high spatial frequencies, we observed a significantly larger Nc amplitude in response to fearful compared to happy faces (*t*_(25)_ = 2.53, *p* = 0.018, *r* = 0.45). In contrast, we did not find a significant difference between the responses to happy and fearful faces when the images contained only LSF (*t*_(25)_ = −0.5, *p* = 0.62, *r* = 0.1). Moreover, we observed a significant difference between LSF and HSF images for happy faces (*t*_(25)_ = −2.65, *p* = 0.014, *r* = 0.47) but not for fearful facial expressions (*t*_(25)_ = 0.05, *p* = 0.96, *r* = 0.01). Specifically, LSF images of happy faces elicited a larger Nc amplitude compared to HSF images of happy faces (LSF: −3.71 ± 1.32 μV (mean ± standard error); HSF: 1.22 ± 1.37 μV), whereas no difference was elicited by HSF when compared to LSF fearful faces (LSF: −2.77 ± 1.66 μV; HSF: −2.84 ± 1.58 μV). In addition, we observed a marginally significant effect of Frequency (*F*_(1,25)_ = 3.77, *p* = 0.063, $\eta_{\text{p}}^{2}$ = 0.13), but no main effect of Emotion (*F*_(1,25)_ = 1.40, *p* = 0.25, $\eta_{\text{p}}^{2}$ = 0.05).

### N290

We did not observe any significant effect between 150 ms and 300 ms at occipital electrodes (Emotion: *F*_(1,25)_ = 1.645, *p* = 0.211, $\eta_{\text{p}}^{2}$ = 0.06; Frequency: *F*_(1,25)_ = 0.676, *p* = 0.419, $\eta_{\text{p}}^{2}$ = 0.03; Emotion*Frequency: *F*_(1,25)_ = 0.177, *p* = 0.677, $\eta_{\text{p}}^{2}$ = 0.01).

### P400

**Figure 3:**
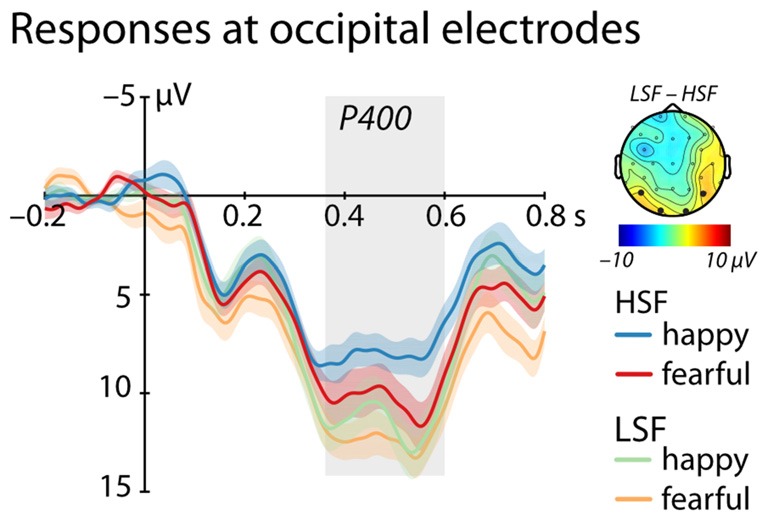
ERP response at occipital electrodes (O1, O2, P7, P8). Shows mean responses at occipital electrodes included in the analysis of the P400 (time-window marked in gray; blue/green = happy expression, red/orange = fearful expression; displayed are mean responses ± within-subject standard errors). The topographic representation show the difference in brain responses to low compared to high spatial frequencies irrespective of emotional expression between 350 ms and 600 ms, corresponding to the time window used in the statistical analysis marked in gray.

Between 350 ms and 600 ms we found a significant main effect of Frequency, (*F*_(1,25)_ = 4.95, *p* = 0.035, $\eta_{\text{p}}^{2}$ = 0.17, see Figure 3), showing a larger P400 amplitude for low compared to HSF faces irrespective of emotional content (LSF: 11.89 ± 1.83 μV; HSF: 9.18 ± 1.92 μV). We did not observe any other significant effects (all *ps* > 0.20).

## Discussion

The current study examined the differential contribution of high and low spatial frequencies to facial emotion processing in 7-month-old infants. Our results show that infants’ brains discriminate between fearful and happy faces only when facial images contain HSF information but not when containing LSF information. This difference is reflected in the modulation of the Nc, which is a neural correlate of attention allocation in infants (de Haan et al., 2003; Webb et al., 2005), suggesting the differential attention allocation relies on detailed information contained in HSF images of these facial expressions. This finding is in line with existing developmental ERP research on this topic from older infants and children (Vlamings et al., 2010; Munsters et al., 2017), also showing that HSF is critical for facial emotion discrimination.

Our analysis further showed that differential processing of HSF emotional faces is driven by the impact of spatial frequency content on the processing of happy faces. This is because the Nc only differed between HSF and LSF happy faces but not between HSF and LSF fearful faces. In other words, the current data suggest that fearful faces are robustly detected regardless of the frequency information contained in the facial stimulus. In contrast, our data show that a smaller Nc amplitude in response to happy faces compared to fearful faces, as commonly reported for unfiltered faces (e.g., Peltola et al., 2009; Grossmann et al., 2011), is only seen when HSF information is presented and disappears when only LSF information is presented. This may point to the importance of detailed information predominantly conveyed via HSF information, presumably from the mouth region (see e.g., Wegrzyn et al., 2017), in eliciting the response typically observed to happy faces at 7 months of age.

Our findings principally agree with Munsters et al. (2017) results from slightly older infants, 9–10 months of age, who also reported specific ERP differences between processing HSF happy and fearful faces. However, while Munsters et al. (2017) observed an emotion effect at the P400 and N290, they did not report an interaction between spatial frequency and emotional content at the Nc as obtained in the current study. One possible reason for these differences across infant ERP studies might be differences in the cut-off used to define low and high spatial frequencies content (>2 cycles/° for LSF images and >6 cycles/° for HSF images by Munsters et al. (2017) as opposed to <0.4 cycles/° for LSF images and >0.6 cycles/° for HSF images in the present study). In this context, it is important to note that our frequency cut-offs were selected on the basis of infant visual acuity at this age, whereas cut-offs chosen by Munsters et al. (2017) were the same as used with adults. Therefore, a direct comparison between studies is problematic since our HSF and LSF range would both be considered as LSF according to Munsters et al. (2017). Furthermore, Munsters et al. (2017) investigated an older age group (9–10 months as opposed to 7 months in the present study) and used a more diverse set of facial stimuli (three emotional facial expressions, faces from different ethnicities, and male as well as female faces as opposed to two emotional expressions from female Caucasian faces only). Especially the use of other-race faces 40% of the time during stimulus presentations in Munsters et al. (2017) study might have influenced infants’ emotion processing since at this age infants have been shown to have difficulty in emotion discrimination from other-race faces (Vogel et al., 2012). Dealing with unfamiliar or less familiar other-race faces might have required them to rely more on an analysis of facial details based on HSF information, which may be reflected at the P400 rather than the Nc. Clearly, future work is needed that directly assesses the exact parameters that impact facial emotion processing when manipulating spatial frequency contents.

The current ERP data show that an enhanced Nc response to fearful when compared to happy faces only occurs for HSF filtered facial stimuli. This pattern obtained for HSF is in line with what has been commonly reported in response to fearful and happy faces using naturalistic photographic images containing the entire frequency range (e.g., Peltola et al., 2009; Grossmann et al., 2011). This suggests that, at 7 months of age, infants rely on HSF information when discriminating between fear and happiness. More specifically, our analyses indicate that HSF happy faces elicit the typical attenuated Nc response seen in previous studies using unfiltered photographs. This might be explained by a need for detailed feature-based information from the mouth region, characteristic for happy faces, for the discrimination to occur (see e.g., Wegrzyn et al., 2017). Alternatively, happy faces might be more difficult to recognize in the LSF condition, leading to increased attention (i.e., larger Nc) as this stimuli may be perceived as slightly ambiguous. In the HSF condition happy faces might be recognized more easily, leading to the typical Nc response. Importantly, our results for the Nc further show that processing fearful faces is immune to the spatial frequency manipulation, suggesting a robust processing of this emotion from the face independent of the specific information contained in the facial stimulus. This further strengthens the notion that fearful faces are effectively detected by infants of this age (Peltola et al., 2009; Jessen and Grossmann, 2016). One potential factor contributing to the robust detection of fearful faces may be infants’ sensitivity to enlarged eye whites, which is known to play a key role in fear perception (e.g., Whalen et al., 2004; Jessen and Grossmann, 2014) and might not be affected by spatial frequency content.

How these findings relate to previous research using functional resonance imaging (fMRI) to track subcortical activity with adults is unclear since the EEG signal is primarily generated by cortical sources (Jackson and Bolger, 2014). Therefore, one way to directly examine the contribution of subcortical regions to emotional face processing in infancy is to resort to fMRI, which has very recently been successfully used to map high-level visual cortical regions implicated in face processing in infants of a similar age (Deen et al., 2017). Another promising approach to use with infants in future studies in order to address the issue of subcortical involvement is to measure pupil dilation, which is primarily subcortically mediated (Bradley et al., 2008) and has been successfully applied to study emotion processing in infants (Hepach and Westermann, 2013; Jessen et al., 2016).

The current data further revealed an enhanced P400 at posterior electrodes in response to LSF compared to HSF faces irrespective of emotional content. The P400 is commonly linked to the processing of structural facial information and is thought to represent the infant precursor to the highly face-sensitive N170 seen in adults (de Haan et al., 2003). Our findings are therefore in agreement with prior empirical work showing that face encoding in the infant brain is mainly driven by LSF (see de Heering et al., 2008) and occurs irrespective of emotional content. Importantly, this pattern further indicates that the spatial filtering applied to our face stimuli was effective in splitting the power spectrum into ranges that are processed differentially by infants because a face-sensitive ERP response, the P400, systematically differed as a function of the spatial frequency. Furthermore, it is critical to mention that the P400 responses elicited in the current study, while smaller in amplitude to HSF faces, were also elicited in response to HSF images, demonstrating that filtering did not abolish or disrupt face-sensitive processing in infants.

In our ERP analysis we did not observe differential processing of emotional faces for either frequency range on the P400, which is in contrast to previous ERP research on this topic with infants (Munsters et al., 2017). In this context, it is important to again mention that there were several methodological differences outlined above that might have contributed to this difference between the current study and previous infant work. First and foremost, in the current study the spatial frequency filters were adjusted to the visual acuity of infants at this age, resulting in largely different HSF and LSF filter ranges. Moreover, emotion effects at the P400 have been observed less robustly compared to emotion effects at other ERP components, especially the Nc. Specifically, some studies report a larger P400 amplitude for fearful compared to happy faces (e.g., Leppänen et al., 2007), whereas other studies did not find a differentiation for this component (e.g., Vanderwert et al., 2015). In summary, the observed P400 effect demonstrates that the HSF and LSF faces used in the current study elicited systematic differences in face-sensitive processes in infants.

## Conclusion

In conclusion, the current study critically adds to our understanding of the neurodevelopment of facial emotion processing in early ontogeny. Our ERP results show that 7-month-old infants distinguish between happy and fearful facial expressions when containing HSF information as reflected in the Nc, suggesting that detailed information matters for this distinction to emerge. This discriminatory ERP effect is driven by an attenuation of the Nc in response to HSF happy faces, whereas the Nc to fearful faces was unaffected by the frequency manipulation. Our results thus provide new insights into the role that spatial frequency information plays when processing facial emotions in infancy, highlighting the robustness of fearful face detection from early in development.

## Author Contributions

SJ and TG designed the study, SJ collected the data, SJ and TG analyzed the data and wrote the manuscript.

## Conflict of Interest Statement

The authors declare that the research was conducted in the absence of any commercial or financial relationships that could be construed as a potential conflict of interest.

## Footnotes

^1^http://www.perceptionweb.com/perception/misc/p271141/pvdmatl.txt
